# Compact Carbon-Based Membrane Reactors for the Intensified Anaerobic Decolorization of Dye Effluents

**DOI:** 10.3390/membranes12020174

**Published:** 2022-02-01

**Authors:** Mohammad Shaiful Alam Amin, Frank Stüber, Jaume Giralt, Agustin Fortuny, Azael Fabregat, Josep Font

**Affiliations:** 1 Departament d’Enginyeria Química, Universitat Rovira i Virgili, Av. Països Catalans 26, 43007 Tarragona, Spain; mohammad.amin@urv.cat (M.S.A.A.); frankerich.stuber@urv.cat (F.S.); jaume.giralt@urv.cat (J.G.); azael.fabregat@urv.cat (A.F.); 2 Department of Chemical Engineering and Polymer Science, Shahjalal University of Science and Technology, Sylhet 3114, Bangladesh; 3 Departament d’Enginyeria Química, Universitat Politècnica de Catalunya, EUPVG, Av. Víctor Balaguer, s/n, 08800 Vilanova i la Geltrú, Spain; agustin.fortuny@upc.edu

**Keywords:** anaerobic decolorization, dye effluents, graphene oxide membrane, matrimid membrane

## Abstract

Carbon-based membranes integrated with anaerobic biodegradation are presented as a unique wastewater treatment approach to deal with dye effluents. This study explores the scope of ceramic-supported carbon membrane bioreactors (B-CSCM) and ceramic-supported graphene oxide membrane bioreactors (B-CSGOM) to decolorize azo dye mixtures (ADM) and other dyes. The mixture was prepared using an equimolar composition of monoazo Acid Orange 7, diazo Reactive Black 5, and triazo Direct Blue 71 dye aqueous solution. Afterwards, as in the ADM experiment, both compact units were investigated for their ability in the biodecolorization of Methylene Blue (MB) and Rhodamine B (RhB) dye solutions, which do not belong to the azo family. The obtained outcomes revealed that the conductive surface of the graphene oxide (GO) membrane resulted in a more efficient and higher color removal of all dye solutions than B-CSCM under a wide feed concentration and permeate flux ranges. The maximum color removal at low feed concentration (50 mg·L^−1^) and permeate flux (0.05 L·m^−2^·h^−1^) was 96% for ADM, 98% for MB and 94% for RhB, whereas it was 89%, 94% and 66%, respectively, for B-CSCM. This suggests that the robust, cost-effective, efficient nanostructures of B-CSGOM can successfully remove diverse azo dye solutions from wastewater better than the B-CSCM does.

## 1. Introduction

Dye molecules are widely applied for coloring in various sectors such as the textile, pulp and paper, leather, drug, food and cosmetics industries. Their use has increased rapidly in recent decades, resulting in massive discharges of toxic, mutagenic, carcinogenic and mostly recalcitrant dye effluents into the environment [1]. The presence of this dyestuff waste in industrial effluent has a detrimental effect on the ecosystem, the human body and animals [2]. The dyed waste turns the color of the water into an unpleasant hue that negatively impacts the photosynthesis process and the amount of dissolved oxygen in aquatic life [3,4]. Moreover, dye-containing wastewater makes water sources unsuitable for drinking and for household and industrial purposes [5]. As a result, developing a simple, economical and sustainable wastewater treatment for removing dye-containing effluent from the environment is considered a critical issue.

At present, several physical, chemical and biological wastewater treatment methods such as adsorption, photocatalysis, coagulation, electrocoagulation, advanced oxidation, ozonation, filtration processes and membrane bioreactors have been examined extensively [6,7,8]. Since most dyestuff molecules are chemically stable and notably soluble in water, it is difficult to remove them using the traditional treatment process [9,10]. In addition, most of these treatment procedures become less appealing due to the higher installation and operating cost, moderate efficiency, duration of the treatment and evolving secondary pollution [11,12]. What is more, this secondary solid waste might be toxic in nature and poses additional environmental problems associated with sludge treatment and disposal. The dye removal process using a membrane is one of the most convenient and cost-effective applications. Membrane units are capable of overcoming some of the previously described limits, such as reduced equipment size, lower energy usage and lower capital costs [13,14]. Additionally, they may eliminate the need for chemicals, making them an environmentally friendly and accessible choice. Among the membrane separation processes, ultrafiltration and nanofiltration are the most used for dye removal; however, fouling limits the membrane flux as well as its performance [11]. In recent years, reverse osmosis (RO) filtration followed by ultrafiltration or nanofiltration has proven effective in removing dye molecules; nevertheless, installation cost remains a significant challenge for its wide application. Electrodialysis and ion-exchange membranes, on the other hand, are also tested for the treatment of water and wastewater.

Due to their effective performance when addressing suspended solids, color and high Biochemical Oxygen Demand (BOD) loads, biological treatment processes are widely used in Wastewater Treatment Plants (WWTPs). These processes can occur in the presence or absence of oxygen (aerobic and anaerobic conditions, respectively). Compared to aerobic treatment, anaerobic biodegradation is a simple and less expensive azo dye decolorization approach [15]. However, in this process, the electron transport between the microorganisms and the dye molecules become the limiting steps that reduce the biodegradability and increase the residence time. Therefore, when searching for an environmentally friendly treatment process for decolorizing dyes from wastewater, it seems that the combination of anaerobic biodegradation and membrane separation processes would be perfectly complementary.

Most of the materials employed for membrane separation used in wastewater treatment are essentially non-conductive in nature [16]. However, a faster electron transfer mechanism in the anaerobic dye reduction is the crux for an effective treatment method [17]. Daniela et al. [18,19] reported that conductive graphene-based composite ion-change membranes could be used for wastewater treatment purposes. Nonetheless, they may not be feasible in terms of cost, which could cause incompetence for large-scale operations with practical applications. Therefore, besides the good mechanical and chemical stability, carbon-based membranes exhibit a conductive layer on the top of their surface that can be integrated with the anaerobic process for dye decolorization [20,21]. Carbon membranes are usually derived from the carbonization of organic polymer materials such as polyimide, cellulose, melamine, polyethersulfone, polyacrylonitrile and the like, in an inert atmosphere [22,23]. A nanostructured carbon membrane for wastewater treatment shows thermally and electrically conductive properties synthesized by graphitic oxide and carbon nanotubes [24,25]. In our previous work, we synthesized two types of ceramic-supported carbon membranes [21,26]. One of the membranes was fabricated by the carbonization of Matrimid 5218 and another one by the vacuum filtration of exfoliated graphene oxide solution. Notably, both the Ceramic Supported Matrimid Membrane (CSCM) and the Ceramic Supported Graphene Oxide Membrane (CSGOM) displayed good electron conductivity that integrated the membrane separation with the anaerobic biodegradation to achieve a successful azo dye decolorization process.

To the best of our knowledge, it is the first time that the removal of azo dye mixture (ADM), phenothiazine Methylene Blue (MB) and sticky fluorescence Rhodamine B (RhB) solutions were conducted in an integrated membrane filtration with an anaerobic biodegradation process. For this, two compact membrane bioreactors using carbonized Matrimid and graphene oxide membranes were studied to decolorize the dye molecules. These two carbon-based membranes were compared to select a more suitable configuration for the anaerobic biodecolorization of azo dye wastewater. Throughout the experiments, the decolorization rate of dye solution was examined under several feed concentrations and permeate fluxes.

## 2. Materials and Methods

### 2.1. Fabrication of Ceramic-Supported Carbon Membrane

The carbon-based membranes were synthesized from several membrane precursors to compare their suitability for the biodecolorization of dyestuff molecules. For this purpose, two membranes were prepared over porous ceramic support (ZrO_2_-TiO_2_ microfiltration flat element, diameter: 47 mm; thickness: 2.5 mm; molecular weight cut-off: 50 kg·mol^−1^; TAMI Industries, Nyons, France) using either a 10% wt. of Matrimid 5218 (Huntsman Advanced Materials, The Woodlands, TX, USA) in NMP (1-methyl-2-pyrrolidone, Sigma Aldrich, ref. 328634, Madrid, Spain), or a 1 mg·mL^−1^ graphene oxide solution, respectively. In CSCM, the polymeric solution was deposited on the support by a two-step spin coating method. After 24 h of drying, the carbon membrane was finally formed by carbonizing it at 800 °C under an inert atmosphere [21]. For the other carbon-based membrane (CSGOM), the exfoliated graphene oxide solution was prepared by the modified Hummer method using 20 μm of pristine graphite powder (Sigma Aldrich, ref. 282863, Madrid, Spain) [27]. The porous graphene oxide layer was deposited over the ceramic support by vacuum-assisted filtration of 3–5 mL of 1 mg·mL^−1^ graphene oxide solution using the filtration cell [26]. The desired thickness of the uniform GO layer was formed within 15 min of vacuum filtration. The membrane was subsequently dried at 80 °C for 24 h and subsequently 100 °C for 72 h to get a stable and robust CSGOM.

### 2.2. Experimental Set-Up for Anaerobic Biodegradation

**Figure 1:**
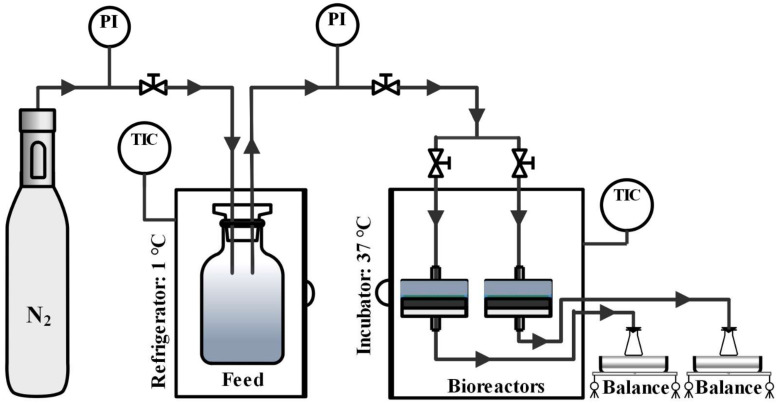
Experimental set-up for anaerobic decolorization of dye molecules by CSCM and CSGOM.

Anaerobic biodecolorization of the dyestuff molecule by carbon-based membrane bioreactor (B-CSCM and B-CSGOM) process is depicted in Figure 1. Nitrogen gas (Purity > 99.99%, Linde, Valencia, Spain) flowed in the feed tank to pump the feed solution to the two membrane bioreactors and modulated the permeate flux by changing the transmembrane pressure (TMP). In addition, it was used to maintain the anaerobic conditions throughout the decolorization process. Thus, the entire system achieves a negative redox potential that forces the suitable conditions for the action of anaerobic microorganisms [28]. The feed solution was composed of dye, Sodium Acetate (Sigma Aldrich, ref. 110191, Madrid, Spain), and basal media. The concentration of Sodium acetate was three times (1:3 molar ratio) higher than the dye in each feed solution. Depending on the test, three different types of feed solutions were used as synthetic feed. The first feed was an equimolar solution of monoazo AO7 (ACROS Organics, ref. 416561000, Geel, Belgium), diazo RB5 (Sigma Aldrich, ref. 306452, Madrid, Spain), and triazo DB71 (Sigma Aldrich, ref. 212407, Madrid, Spain). The second and third were the solution of Methylene Blue (Fluka, ref. 66720, Madrid, Spain) and Rhodamine B (Fluka, ref. R6626, Madrid, Spain), respectively. On the other hand were basal media with microelements (mg·L^−1^) composed by MnSO_4_·H_2_O (0.155), CuSO_4_·5H_2_O (0.285), ZnSO_4_·7 H_2_O (0.46), CoCl_2_·6H_2_O (0.26), (NH_4_)_6_Mo_7_O_24_ (0.285), K_2_HPO_4_ (21.75), Na_2_HPO_4_·2H_2_O (33.40), KH_2_PO_4_ (8.50), FeCl_3_·6H_2_O (29.06), CaCl_2_ (13.48) MgSO_4_·7H_2_O (15.2), NH_4_Cl (190.90). All the analytical grade chemicals were purchased from Sigma Aldrich (Madrid, Spain) and dissolved in Milli-Q water (Millipore Milli-Q system, Molsheim, France).

Temperature has a significant impact on decolorization performance and microbial population in this compact membrane bioreactor. Thus, the feed solution was kept at 1 °C to prevent microbial growth on the feed tank and maintain its actual dye and co-substrate (Sodium Acetate) composition. After placing 5 mL of secondary anaerobic sludge (collected from municipal WWTP Reus, Spain) over the carbon-based membrane, The membrane bioreactor, which was a direct-filtration cell (TAMI Industries, Nyons, France), was sealed tightly and then operated at 37 ± 1 °C under constant flux dead-end filtration mode. At this temperature, the microbial strains are able to give the best biodecolorization rate [29].

### 2.3. Membrane Characterization

The Combined Focused Ion Beam-Scanning Electron Microscope (FIB-SEM, Scios 2 Dual Beam, Thermo Scientific, MA, USA) examined and compared the surface, cross-section and elemental composition of both CSCM and CSGOM. An Atomic Force Microscope (AFM) using Molecular Imaging Pico Plus 2500 (Bid Service, NJ, USA) examined the membrane surface and conductivity. Furthermore, chemical structures and microstructures of the carbon-based membranes were analyzed, respectively, through a Fourier Transform Infrared (FT-IR) spectrophotometer (FT/IR-6700, JASCO, Tokyo, Japan) and an X-ray Diffraction (XRD) diffractometer (Siemens D5000, Germany under the CuKα wavelength of 1.54056 Å at 40 kV and 30 mA), respectively.

The filtration performance was assessed by means of membrane flux, pure water permeability (PWP) and hydraulic resistance, and calculated using Equations (1)–(3),

(1) $$J=\frac{\Delta V}{\Delta t}\cdot \frac{1}{A}\;$$

(2) $$\mathrm{PWP}=\frac{J}{\Delta P}$$

(3) $$H_{R}=\frac{\Delta P}{\mu }\cdot \frac{1}{J}$$

where ***J*** is the permeate flux (L·m^−2^·h^−1^), *V* is the volume of permeate (L), *t* the filtration time (h), *A* the membrane area (m^2^), *H_R_* is the hydraulic resistance (m^−1^), *∆P* is the transmembrane pressure (bar) and *μ* is the viscosity of the permeate corrected to the experimental temperature (Pa·s).

The decolorization (***D***) during the anaerobic bioreduction of the azo dye was measured using UV/VIS4000n Spectrophotometers (DINKO Instruments, Barcelona, Spain) and computed by Equation (4),

(4) $$D\;(\%)=\frac{A_{o}-A}{A_{o}}\times 100$$

*Ao* and *A* are the absorbance of feed and treated samples during the biodegradation process, respectively.

## 3. Result and Discussion

### 3.1. Structural and Chemical Characterization of Ceramic-Supported Carbon Membranes

**Figure 2:**
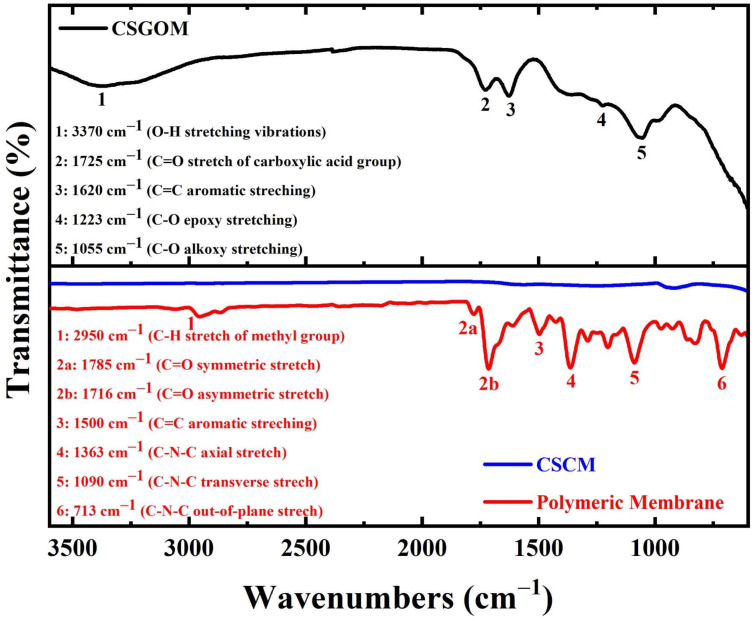
FTIR spectra of the polymeric membrane, CSCM and CSGOM (CSCM: 10% wt. Matrimid and CSGOM: 1 mg·L^−1^ GO solution).

The FTIR spectrum of carbon-based membranes shown in Figure 2 helps to reveal the presence of diverse functional groups, including carboxylic, hydroxyl, epoxy and imide, that also exhibited the appropriate molecular orientation. For the polymeric membrane and CSCM, before and after the carbonization of the membrane, the structural changes were observed through the transformation of the polymeric chains to the carbon membrane. The representative bands of the polymeric membrane (Matrimid 5218) were obtained at 1714 and 1360 cm^−1^, indicating the stretching of C=O and C-N groups [30]. The aromatic C=C bending vibration, C-N-C transverse stretching and presence of C-H aromatic monosubstituted benzene were found at 1501, 1089 and 718 cm^−1^, respectively [31]. In the case of CSCM, compared to the initial polymeric membrane, no apparent bands were observed after its carbonization under a nitrogen atmosphere. This fact was similar to that observed by Sazali et al. [32], where they synthesized the Matrimid-based carbon membrane at different pyrolysis temperatures and polymeric compositions.

The peaks for numerous oxygen-containing functional groups, such as O-H, C=O and C-O, were visible in the CSGOM spectra, which agrees well with earlier research and illustrates that the graphite exists mostly oxidized into GO [33,34]. In summary, the broad peak at 3370 cm^−1^ confirms the presence of hydroxyl groups on the GO layer of CSGOM while the peak at 1725 cm^−1^ indicates the C=O stretching vibration of carboxylic acid positioned at the edge of the GO structure. The absorption peaks at 1620, 1223 and 1055 cm^−1^, respectively, were obtained due to the aromatic C=C stretching, epoxy and alkoxy C-O stretching vibration.

**Figure 3:**
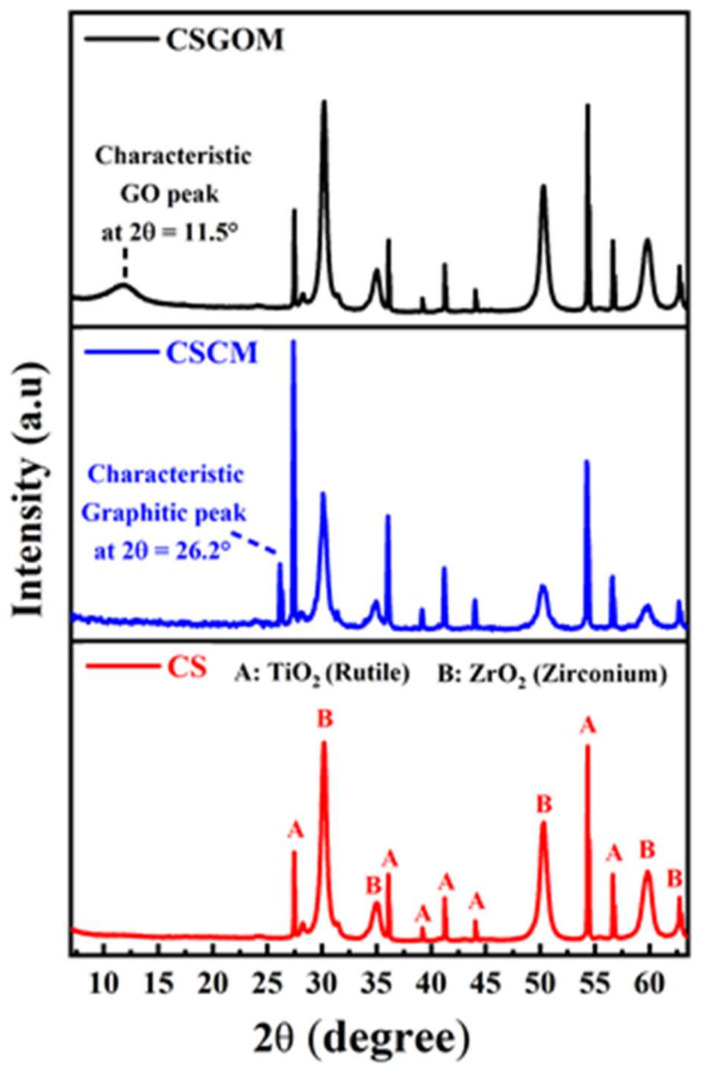
X-ray diffractogram of the ceramic support (CS) and the ceramic-supported carbon-based membrane (CSCM: 10% wt. Matrimid and CSGOM: 1 mg·L^−1^ GO solution).

The microstructure and interlayer distance of the ceramic support and ceramic supported carbon-based membranes were determined using XRD diffraction, as shown in Figure 3. In CS, CSCM and CSGOM diffractograms, identical peaks were obtained at 2θ = 28, 36, 39, 41, 43, 55 and 57° due to the presence of rutile, TiO_2_ [35], and 2θ = 30, 35, 50, 60, and 63° due to the presence of tetragonal ZrO_2_ [36], which is consistent with the information provided by the supplier of the ceramic support, consisting of layers of ZrO_2_ and TiO_2_. Furthermore, the diffraction peak located at 2θ = 26.2° is an indication that the CSCM consisted of highly graphitized carbon with an interlayer distance of 3.6 Å, which corresponds to the (002) plane of crystalline graphite [37]. In addition, the GO crystal plane (001) was clearly visible at 2θ = 11.5° (as shown in CSGOM diffractogram in Figure 3) with the interlayer spacing of 7.3 Å that was larger than graphite atomic spacing [38]. The absence of a peak at 26.2° means that all graphite molecules were completely oxidized to generate graphene oxide, and no graphitic contaminations were obtained [39]. This was expected as the oxygen-containing groups were attached to each layer edge to raise the spacing between the sheets, which aided GO exfoliation in the aqueous medium and increased the hydrophilicity [40].

**Figure 4:**
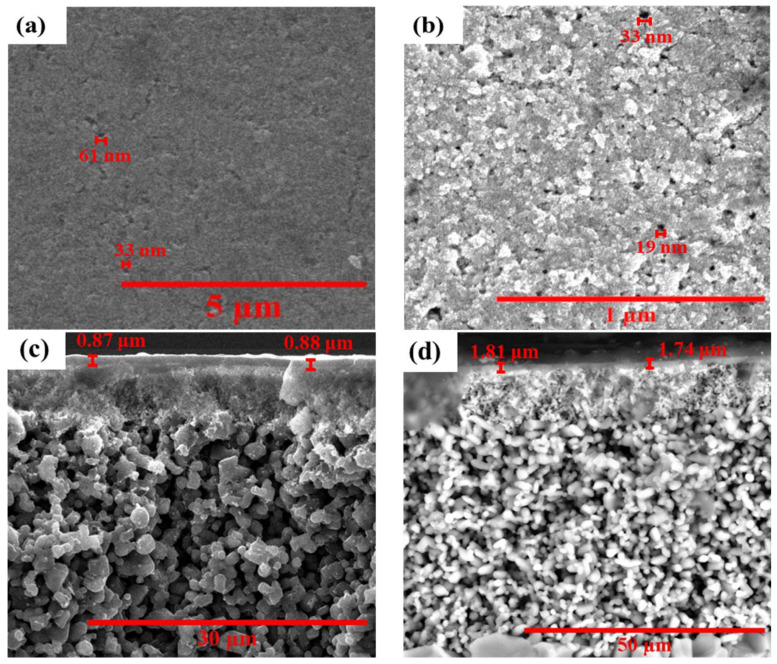
FESEM micrographs on the surface of (**a**,**b**) CSCM and CSGOM, and (**c**,**d**) cross section of CSCM and CSGOM (CSCM: 10% wt. Matrimid and CSGOM: 1 mg·L^−1^ GO solution).

The surface and cross-sectional views of the carbon-based membranes were studied using FESEM, which shows crucial morphological features for their application in the anaerobic decolorization process. As illustrated in Figure 4a,b, the top view of these carbon membranes clearly exhibits a defect-free smooth and porous carbon layer that was quite similar to previous studies [21,26]. The pore size of the resulting carbon membrane differed between the two alternatives. The difference between both membranes was the pore size and thickness of the respective synthesized carbon layers. The average pore size was found to be 35 μm for CSCM and 20 μm for CSGOM. On the other hand, the thickness was, respectively, 0.88 and 1.78 μm for CSCM and CSGOM, as depicted in Figure 4c,d. The thinner layer in CSCM was due to the optimal spin coating method and high carbonization temperature maintained during the preparation of this membrane [41]. For the same reason, FESEM-EDX analysis observed higher carbon content in CSCM (64%) than that of the CSGOM (49%).

**Figure 5:**
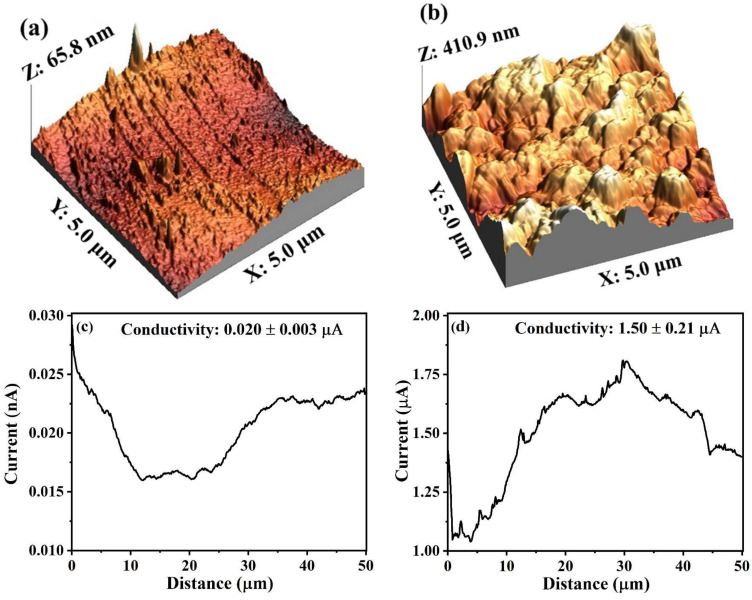
AFM images of CSCM and CSGOM (**a**,**b**) 3D Topography and (**c**,**d**) current distribution.

The membrane surface roughness and conductivity were further investigated using AFM (at a random area of 5 × 5 μm^2^) and CSAFM (current sensing atomic microscopy) images. The 3D topography and current profile of these experiments are shown in Figure 5a–d. The root-mean-square (RMS) roughness of the carbon layer attained for the CSGOM surface was at 80.12 nm, while the CSCM exhibited the lowest surface roughness at 32.94 nm. This fact was attributed to the less porous structure of the carbonized Matrimid membrane, as shown by SEM images. This means a less porous structure but represents a smoother surface. In Figure 5c,d, the current distribution of the surface of the membrane was compared, and the local conductance on the graphene oxide membrane surfaces was found better than that of the CSCM. However, the rough conductive GO surfaces could improve the biodecolorization because of the more vigorous attachment of the biofilm to the surface and the intensified electron shuttle mechanism [42,43].

Therefore, it is possible to conclude from the above results that the compact membrane bioreactor made with a graphene oxide layer may be a more suitable solution for the dye bioreduction method.

### 3.2. Impact of the Carbonaceous Layer on Flux and Resistance

**Figure 6:**
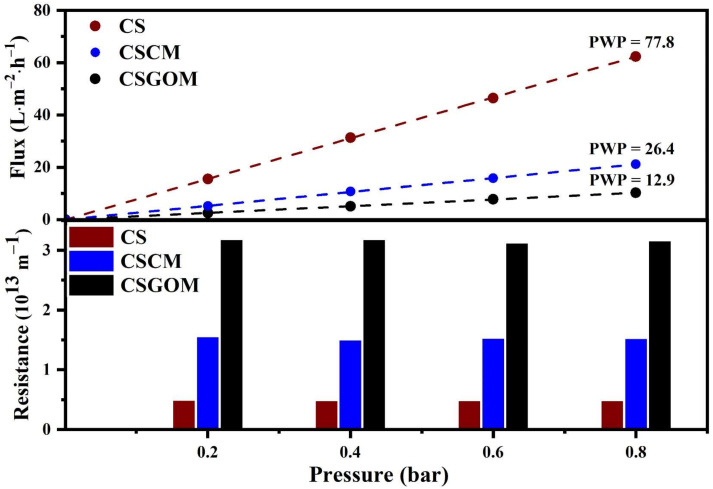
Variation of pure water flux, permeability, and resistance of CS, CSCM and CSGOM at 25 °C. CSCM: 10% wt. of Matrimid and CSGOM: 1 mg·mL^−1^ of GO.

The effect of membrane precursors (Matrimid 5218 or exfoliated GO solution) on filtration parameters such as permeate flux, pure water permeability (PWP) and hydraulic resistance (*H_R_*) was examined by experiment. Figure 6 displays the results of this comparative study, where the units were either the ceramic support (CS), CSCM (produced with 10% wt. of Matrimid solution) or CSGOM (made with 1 mg·mL^−1^ of exfoliated GO solution). A simple comparison shows that the ceramic support gives the maximum permeate flux, 62.3 L·m^−2^·h^−1^, and 77.8 L·m^−2^·h^−1^·bar^−1^ of PWP. At the same range of operating conditions, the lowest water flux (10.2 L·m^−2^·h^−1^) and PWP (12.9 L·m^−2^·h^−1^·bar^−1^) were observed for CSGOM, which is less than 84% and 50%, respectively, than for CS and CSCM. In addition, a bar chart depicting the hydraulic resistance of the CS, CSCM and CSGOM is shown at the bottom of Figure 6. Since both variables are directly related, CS displayed the lowest hydraulic resistance (5.25 ± 0.1 × 10^12^ m^−1^), while CSGOM has the highest.

The effect of membrane precursors (Matrimid 5218 or exfoliated GO solution) on filtration parameters such as permeate flux, pure water permeability (PWP) and hydraulic resistance (***H_R_***) was examined by experiment. Figure 6 displays the results of this comparative study, where the units were either the ceramic support (CS), CSCM (produced with 10% wt. of Matrimid solution) or CSGOM (made with 1 mg·mL^−1^ of exfoliated GO solution). A simple comparison shows that the ceramic support gives the maximum permeate flux, 62.3 L·m^−2^·h^−1^, and 77.8 L·m^−2^·h^−1^·bar^−1^ of PWP. At the same range of operating conditions, the lowest water flux (10.2 L·m^−2^·h^−1^) and PWP (12.9 L·m^−2^·h^−1^·bar^−1^) were observed for CSGOM, which is less than 84% and 50%, respectively, than for CS and CSCM. In addition, a bar chart depicting the hydraulic resistance of the CS, CSCM and CSGOM is shown at the bottom of Figure 6. Since both variables are directly related, CS displayed the lowest hydraulic resistance (5.25 ± 0.1 × 10^12^ m^−1^), while CSGOM has the highest.

Membrane thickness and pore size greatly influence the membrane performance by altering the permeate flux and hydraulic resistance [44]. In this study, in addition to the TiO_2_-ZrO_2_ layers of the ceramic support, we deposited an additional carbon layer on top of the support in each case. As a result, not only does the permeability of the membrane drops but the hydraulic resistance rises. Comparing the FESEM images of the two membranes synthesized over the ceramic support, the thickness of the defect-free carbon layer in CSCM was found to be smaller than that of CSGOM; on the contrary, the average pore size was relatively greater. Thus, the initial PWP (77.8 L·m^−2^·h^−1^·bar^−1^) and resistance (5.25 ± 0.1 × 10^12^ m^−1^) of the porous ceramic support changed after deposition of the carbonaceous layer. Due to the relatively larger thickness and smaller pore size of CSGOM than CSCM, PUP was recorded at 12.9 L·m^−2^·h^−1^·bar^−1^; on the other hand, in CSCM, it was 26.4 L·m^−2^·h^−1^·bar^−1^. Similarly, the hydraulic resistance rises to 1. 50 ± 0.2 × 10^13^ m^−1^ in CSCM and 3.15 ± 0.3 × 10^13^ m^−1^ in CSGOM.

These results align with the trends observed by Sazali et al. [45], which indicated that the high carbonization temperature and precursor concentration were the keys for fabricating a thin carbon-based membrane. In the case of CSGOM, the membrane was prepared by vacuum-assisted filtration of an exfoliated GO solution with a concentration of 1 mg·mL^−1^. The viscosity of the GO solution is much lower than that of Matrimid 5218, so it easily filled the internal ceramic support pore (GO-ZrO_2_ region) and then formed the mono or multilayer of GO on the top of CS. Due to internal blockage or filling of the support pore, the hydraulic resistance and permeability through the membrane were usually disrupted [46].

Our recent work demonstrated the selection of the most appropriate precursor is critical to achieving a porous, permeable carbon-based membrane [21,26]. In CSGOM, this is essential because the carbon layer thickness on the membrane surface increases as the GO concentration increases, resulting in a decrease in membrane flux [47]. Again, when the membrane is derived from a high precursor concentration (>3 mg·mL^−1^), the GO layer can easily peel away from the surface or cause swelling of the layer [48]. On the other hand, GO membranes synthesized with low GO solution (0.05–0.5 mg·mL^−1^) provide low carbon content that was not suitable for anaerobic biodecolorization application. A thicker and less porous GO layer was required to establish a defect-free GO layer with high carbon content and flux on CSGOM. Thus, compared to the CSGOM, pure water flux and permeability values were higher in CSCM. Since permeate flux and hydraulic resistance are interdependent, higher hydraulic resistance was observed in the CSGOM due to its lesser porosity and formation of GO-ZrO_2_ layers inside the ceramic support.

### 3.3. Role of the Membrane Precursors on Anaerobic Decolorization of Dye Molecules

**Figure 7:**
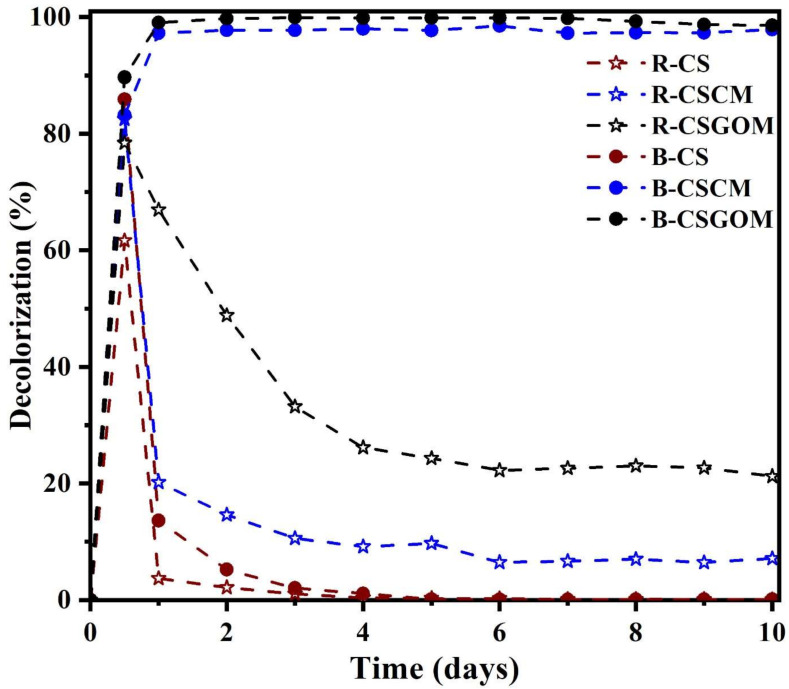
Decolorization of azo dye mixture in CS, CSCM and CSGOM reactors, and CS, CSCM and CSGOM bioreactors. Flux = 0.05 L·m^−2^·h^−1^, [ADM]_o_ = 50 mg·L^−1^ and T = 37 °C.

The combined effect of the filtration and anaerobic bioreduction of dye solutions in compact bioreactors was evaluated using carbon-based membranes (both CSCM and CSGOM) and ceramic support (CS). This study used 50 mg·L^−1^ azo dye mixture (ADM) of feed solution that contained an equimolar mixture of the monoazo AO7, diazo RB5, and triazo DB71. All reactors were operated in dead-end filtration mode and maintained with a constant permeate flux of 0.05 L·m^−2^·h^−1^. These concentrations in the feed solution were selected because they fall within the range owing to dye residues in industrial or textile wastewater, which is generally found between 10 and 50 mg·L^−1^ [49]. In this test, the reactors operated with microorganisms are referred to as B-CS, B-CSCM, and B-CSGOM; in turn, reactors without microorganisms are labeled R-CS, R-CSCM, and R-CSGOM, respectively. As illustrated in Figure 7, the simultaneous presence of the carbon layer and microorganisms has a significant effect on the bioreduction of ADM during the decolorization process. Both compact carbon-based membrane bioreactors, B-CSCM and B-CSGOM, gave very high decolorization rates under anaerobic conditions, with B-CSGOM achieving a maximum of 99% dye removal rate.

At the start of the anaerobic decolorization process, up to 12 h, all the reactors accomplished more than 75% of the dye removal rate. Unlike the two carbon-based membrane bioreactors, the decolorization rate for all the other reactors progressively declined; it is noteworthy that, after 48 h of operation, the color removal for B-CS and R-CS dropped to almost zero. This strongly suggests that the dye was initially removed due to adsorption by the porous support and carbon membrane but, once saturated, they were not able to remove color anymore. Even though B-CS was used in conjunction with microorganisms, the color removal was negligible, too. It seems that the microorganisms were washed out from the reactor by the permeate flow because of the lack of nano-sized structure or support suitable to form a stable biofilm, as happens in the other carbon-based membranes. For CSGOM and CSCM, because of having a nanoporous carbon layer on the top, decolorization gradually decreased to reach a stable 20% for R-CSGOM and 6% for R-CSCM. In this case, the pore size of the carbonaceous layers was able to partially reject the dyes, although both the adsorption site and pore size decreased with the stream time [50]. The highest decolorization, 99%, was observed for the bioreactor operated with CSGOM, while 97% of color removal was observed for B-CSCM. Overall, it means that the carbon layer derived from the carbonization of the Matrimid and graphene oxide has no adverse effect on bacterial growth for the successive formation of active biofilm [51,52,53]. As with the results obtained from the earlier ceramic-supported carbon-based membrane bioreactor experiments [21,26], these membranes perform triple roles in anaerobic dye decolorization. The carbon membrane served as biofilm support, redox mediator, and pollutant immobilizer that also retain the degradation products. In this way, the one-pass compact carbon-based membrane bioreactor has shown enhanced decolorization performance for azo dye mixtures.

Following each successful biodecolorization experiment, the membrane was cleaned using Milli-Q water backflushing. As a result, the initial permeate flux was regained without modifying the membrane properties, so it can be reused for subsequent color removal operation without any additional conditioning.

### 3.4. Biodecolorization Performance of CSCM and CSGOM

After demonstration of stable and excellent performance by the carbon-based membrane bioreactors, the B-CSCM and B-CSGOM were investigated for long-term operation for the biodecolorization of structurally different dyes. Azo dye mixture, thiazine MB and fluoresce RhB dye solutions were examined under various permeate fluxes and feed concentrations. Initially, all compact reactors were run with 50 mg·L^−1^ of dye solution at a permeate flux of 0.05 L·m^−2^·h^−1^. After obtaining consistent decolorization, the system was left to operate for 10 days; then, the flow rate was first increased to 0.075 L·m^−2^·h^−1^ and, finally, at 20 days, up to 0.1 L·m^−2^·h^−1^. Similar procedures were repeated for compact bioreactors consisting of 75 and 100 mg·L^−1^ of feed solution operated for the anaerobic decolorization of azo dyes.

**Figure 8:**
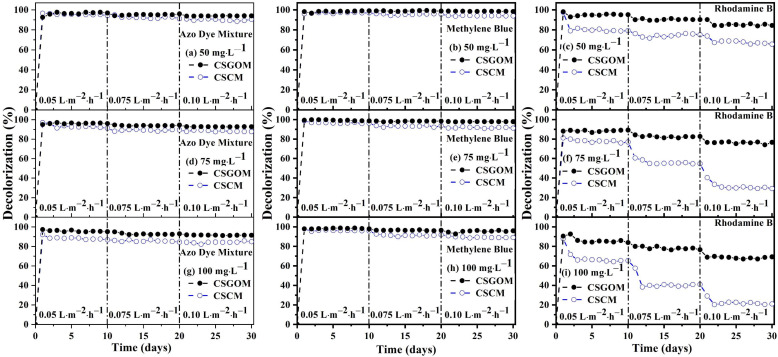
Anaerobic decolorization of the Azo dye mixture, Methylene Blue, and Rhodamine B dyes at various concentrations and fluxes; (**a**–**c**) 50 mg·L^−1^, (**d**–**f**) 75 mg·L^−1^ and (**g**–**i**) 100 mg·L^−1^ at 37 °C. CSCM: 10% wt. of Matrimid and CSGOM: 1 mg·mL^−1^ of GO.

Figure 8 collects all the decolorization performance data. Besides the influence of the feed concentration and permeate flow, it is shown that the biodecolorization effectiveness of different dye solutions widely depends on the number and nature of azo bonds and functional groups present in the dye structure. Apart from this, membrane properties, such as membrane precursors, pore size, and thickness, also played a relevant role in decolorization performance. It must be noted that all the dye decolorization removals decreased when feed concentration and permeate flux increased. In all experiments, the highest decolorization was achieved for the lowest feed concentration and permeate flow. Comparing again the performance of the bioreactors containing carbon-based membranes, B-CSGOM exhibits greater decolorization under all feed concentrations and fluxes. According to Figure 8, the maximum decolorization in the B-CSGOM system was 96%, 98%, and 94% for ADM, MB, and RhB, respectively, (obtained for 50 mg·L^−1^ of dye solutions at a permeate flux of 0.05 L·m^−2^·h^−1^). On the contrary, the lowest decolorization was in B-CSCM experiments using 100 mg·L^−1^ feed solutions and 0.10 L·m^−2^·h^−1^ flow, which only achieved 84% for ADM, 84% for MB and 22% for RhB.

In terms of lifetime, the B-CSGOM was more stable in all operating situations and produced better results. This indicates that the properties of the membrane precursor were responsible for most of the dye removal capacity in the carbon-based membrane bioreactors operated under identical conditions. Thus, in addition to providing the required energy for microbial growth, it works as a redox mediator by enhancing the electron transfer to the azo, hydrogen and other bonds of the dye molecule, thereby breaking the bonds to achieve the effective biodegradation of dyes [17].

However, regardless of the flux, the difference of the decolorization between B-CSCM and B-CSGOM was rather low for ADM and MB, whereas this was indeed noticeable for RhB. In general, the color removal efficiency for ADM (96–94% in B-CSGOM and 95–88% in B-CSCM), MB (98% in B-CSGOM and 96% in B-CSCM) and RhB (94–84% in B-CSGOM and 79–66% in B-CSCM) was remarkably steady at low permeate flux, 0.05 L·m^−2^·h^−1^, as illustrated by Figure 8, with no significant steady changes found when the feed concentrations were first increased up to 75 mg·L^−1^ and then up to 100 mg·L^−1^. The results obtained were mostly consistent and, in some cases, showed much better decolorization performance than those using conventional adsorption, photocatalysis, filtration and bacterial treatment processes for decolorizing the dye mixture, Methylene Blue and Rhodamine Blue dye solutions [2,16,54]. It must be noted that dye removal levels reached here were similar to those obtained using nanofiltration coupled with wet oxidation, PVDF membrane sensitized nano zeolite, MBR with electrocoagulation and Ag/Pd-loaded ZnO nanofiber membrane with UV irradiation, where over 85% of color removal [55,56,57,58] was shown. Some other membrane-based dye removal treatment processes, such as nanofiltration under unstirred batch and continuous crossflow mode [59] and activated carbon-PVDF Blend Membrane [60] showed the limitations and were capable of decolorizing just up to 70%.

During the biodecolorization process, the biomass load was sufficient to form active biofilms to ensure complete biodegradation of the dyes, even with the short contact times associated to one-pass direct filtration operation. Even so, low permeate flow allowed the dye molecules to interact sufficiently with the microorganisms, stimulating the effective biodecolorization rate [61,62], though higher permeate flux is of course detrimental for achieving high biodegradation [63,64]. Therefore, the increase of the permeate flux from 0.05 to 0.075 L·m^−2^·h^−1^ and, finally, to 0.1 L·m^−2^·h^−1^ provides declining decolorization of ADM, MB and RhB dye solutions up to 94%, 98% and 85%, respectively, in B-CSGOM, which are more evident for B-CSCM, where they decreased to 89% for ADM, 94% for MB and 66% for the most reluctant RhB.

Increased feed concentration of dye molecules introduced more reactive groups into the anaerobic bioreactor, as did increasing permeate flux. In such conditions, the excess load of functional groups causes an inadequate biomass to dye ratio [16,63]. Therefore, some dye molecules escape the membrane reactor without undergoing adequate biodegradation. Additionally, the toxicity effect of the dye molecules affects active sites in the biofilm, reducing the decolorization effectiveness of the membrane reactor. These features were reflected in Figure 8a–c, where the color removal for each of the dye solutions and compact bioreactors declined as the initial feed concentration or permeate flux increased so the load to the anaerobic biodegradation system rises. The feed concentration was first increased from 50 to 75 mg·L^−1^ and, later, to 100 mg·L^−1^, in B-CSGOM; accordingly, the decolorization rate for the ADM, MB and RhB solutions was reduced to 91%, 96% and 67%, respectively. Whereas for B-CSCM, it was decreased to 84% for ADM, 88% for MB and 22% for RhB. In an earlier investigation, the color removal rate of individually administered AO7, RB5 and BD71 solutions was respectively 34–58% in B-CSCM and 86–97% in B-CSGOM [21,26].

For the azo dye mixture, this study achieved a decolorization up to 90% and 94% for B-CSCM and B-CSGOM, respectively. In the instance of ADM, the mixed solution exhibited a synergic effect, which increased the biodecolorization of the ADM compared to the individual dye. Additionally, the mixture of numerous azo dyes and their metabolic products boosts the carbon sources or food available to the microbe that improves the bioreduction of dye molecules. B-CSGOM, on the other hand, performs significantly better than B-CSCM because its GO layer is more conductive than the CSCM carbon layer to escalate the biodegradation performance.

As can be seen, color removal for the MB exhibited less sensitivity to the bioreactor configuration and operating conditions. This treatment behavior can be influenced by the factors that dye removal efficacy varies, such as dye structure with different bonds and functional groups, dye concentration, external carbon and nitrogen sources, electron donor, redox mediator, food to microorganism ratio, and the microorganism growth [65,66]. Increasing feed concentration of ADM in the bioreactor means more azo bonds but also that more other functional groups were entered into the biodegradation system. On the contrary, the rate of biodecolorization of the RhB dye solution was the lowest in all cases. RhB molecules are coalesced by nature and easily stick into the pores or the biofilm. As a result, activity and development of the microbial were prevented, which reduced the biodegradability performance of RhB. The reason for the two-and-a-half-fold higher rate of RhB decolorization in B-CSGOM in comparison to B-CSCM should be assigned to the best capacity of graphene oxide to transfer electrons, which is critical for this more structurally complex dye.

## 4. Conclusions

In this study, the feasibility of two carbon-based membranes integrated with an anaerobic process proved to be efficient for treating several dye solutions. The systems were tested to decolorize azo dye mixtures made of monoazo Acid Orange 7, diazo Reactive Black 5 and triazo Direct Blue 71 dyes. In addition, they were also tested over the phenothiazine, Methylene Blue and the sticky fluorescence Rhodamine B (RhB).

It was found that the enhanced electron transfer mechanism of graphene oxide in the CSGOM unit seems to be superior to the carbonaceous layer present in the CSCM configuration. Additionally, this nanoscaled GO layer appears to stimulate the establishment of stable biofilms and to retain better dye molecules and biodegradation derived compounds.

Under the lowest feed concentration (50 mg·L^−1^) and permeate flux (0.05 L·m^−2^·h^−1^), maximum decolorization of the dye solutions in CSGOM bioreactor was 96% for ADM, 98% for MB and 94% for RhB; in turn, for B-CSCM, it was 89%, 94% and 66%. In response to increasing feed and flux, after 30 days of operation, the decolorization rate of ADM and MB was only 8–12% and 2–5% lower in B-CSCM and B-CSGOM, respectively. RhB was demonstrated to be more reluctant, so the decolorization lowered up to 67% in B-CSGOM and only 26% in B-CSCM for the more unfavorable conditions, higher feed concentration and flux.

The present investigation shows that compact graphene oxide membrane-based anaerobic biosystems can be successfully applied for the decolorization of a wide variety of isolate or mixed dye molecules.

The definition of the kinetics describing the anaerobic bioreduction of azo dyes is highly needed in order to optimize the decolorization performance so the implementation at a large scale can be lowered. In this sense, operation at higher permeate flux or availability of more conductive membranes would be desirable, too.

## Data Availability

The data presented in this study are available on request from the corresponding author.
